# Supplementary material for: Simultaneous Quantification and Pharmacokinetic Study of Nine Bioactive Components of Orthosiphon stamineus Benth. Extract in Rat Plasma by UHPLC-MS/MS
Source: Molecules. 2019 Aug 22;24(17):3057. doi: 10.3390/molecules24173057 (PMC6749594; doi:10.3390/molecules24173057)
Supplement: Supplementary file 1 [file molecules-24-03057-s001.pdf]

molecules

Simultaneous Quantification and Pharmacokinetic Study of Nine Bioactive Components of *Orthosiphon stamineus* Benth. Extract in Rat Plasma by UHPLC-MS/MS

Zili Guo [1], Bo Li [2], Jinping Gu [2], Peixi Zhu [2], Feng Su [2], Renren Bai [2], Xianrui Liang [2,*] and Yuanyuan Xie [1,2,*]

[1] Key Laboratory for Green Pharmaceutical Technologies and Related Equipment of Ministry of Education, Zhejiang University of Technology, 18 Chaowang Road, Hangzhou 310000, China
[2] College of Pharmaceutical Science, Zhejiang University of Technology, 18 Chaowang Road, Hangzhou 310000, China
* Correspondence: liangxrvicky@zjut.edu.cn (X.L.); xyycz@zjut.edu.cn (Y.X.); Tel.: +86-1895-805-7635 (Y.X.)

Table 1. The mass spectrometric parameters of the analytes.

Analytes	Precursor Ion→Product Ion	CV (V)	CE (V)	Ion Mode
PCA	153.1223→81.0969	2	22	Negative
	153.1223→91.0576	2	24	
DSS	197.1486→135.1198	36	16	Negative
	197.1486→123.0824	36	14	
CAA	179.1380→107.0708	2	24	Negative
	179.1380→89.1040	2	30	
RA	359.1803→161.1187	48	14	Negative
	359.1803→197.0986	48	14	
SIN	373.2166→343.2086	22	26	Positive
	373.2166→312.2431	22	24	
EUP	345.2492→312.1695	12	24	Positive
	345.2492→284.1967	12	28	
CA	473.1117→135.0833	2	58	Negative
	473.1117→311.1818	2	10	
Sal A	493.1532→109.0595	2	30	Negative
	493.1532→295.1926	2	16	
Sal B	717.1853→321.1844	2	34	Negative
	717.1853→519.2154	2	20	
CHL	321.1081→152.0853	56	12	Negative
	321.1081→257.1026	56	10	
